# Benzalkonium tolerance genes and outcome in *Listeria monocytogenes* meningitis

**DOI:** 10.1016/j.cmi.2016.12.008

**Published:** 2017-04

**Authors:** P.H.C. Kremer, J.A. Lees, M.M. Koopmans, B. Ferwerda, A.W.M. Arends, M.M. Feller, K. Schipper, M. Valls Seron, A. van der Ende, M.C. Brouwer, D. van de Beek, S.D. Bentley

**Affiliations:** 1) Department of Neurology, Centre for Infection and Immunity Amsterdam (CINIMA), Academic Medical Centre, University of Amsterdam, Amsterdam, The Netherlands; 2) Pathogen Genomics, Wellcome Trust Sanger Institute, Wellcome Trust Genome Campus, Hinxton, UK; 3) Department of Medical Microbiology, Centre for Infection and Immunity Amsterdam (CINIMA), Academic Medical Centre, University of Amsterdam, Amsterdam, The Netherlands; 4) Netherlands Reference Laboratory for Bacterial Meningitis, Academic Medical Centre/RIVM, University of Amsterdam, Amsterdam, The Netherlands

**Keywords:** Bacterial genetics, Bacterial meningitis, Genome-wide association study, *Listeria monocytogenes*, Meningitis

## Abstract

**Objectives:**

*Listeria monocytogenes* is a food-borne pathogen that can cause meningitis. The listerial genotype ST6 has been linked to increasing rates of unfavourable outcome over time. We investigated listerial genetic variation and the relation with clinical outcome in meningitis.

**Methods:**

We sequenced 96 isolates from adults with listerial meningitis included in two prospective nationwide cohort studies by whole genome sequencing, and evaluated associations between bacterial genetic variation and clinical outcome. We validated these results by screening listerial genotypes of 445 cerebrospinal fluid and blood isolates from patients over a 30-year period from the Dutch national surveillance cohort.

**Results:**

We identified a bacteriophage, phiLMST6 co-occurring with a novel plasmid, pLMST6, in ST6 isolates to be associated with unfavourable outcome in patients (p 2.83e-05). The plasmid carries a benzalkonium chloride tolerance gene, *emrC*, conferring decreased susceptibility to disinfectants used in the food-processing industry. Isolates harbouring *emrC* were growth inhibited at higher levels of benzalkonium chloride (median 60 mg/L versus 15 mg/L; p <0.001), and had higher MICs for amoxicillin and gentamicin compared with isolates without *emrC* (both p <0.001). Transformation of pLMST6 into naive strains led to benzalkonium chloride tolerance and higher MICs for gentamicin.

**Conclusions:**

These results show that a novel plasmid, carrying the efflux transporter *emrC*, is associated with increased incidence of ST6 listerial meningitis in the Netherlands. Suggesting increased disease severity, our findings warrant consideration of disinfectants used in the food-processing industry that select for resistance mechanisms and may, inadvertently, lead to increased risk of poor disease outcome.

## Introduction

Listeriosis is caused by eating food contaminated with the bacterium *Listeria monocytogenes* and poses an important public health problem [1]. In humans it can cause a range of infections including gastroenteritis, bacteraemia, sepsis and meningitis [1]. The decline since the 1990s in incidence of invasive disease caused by *L. monocytogenes* has been attributed to a decrease in contamination through ready-to-eat food following improvements in the food-processing industry [2]. However, food-processing plants have increasingly been implicated as an infection source of listeriosis [3]. The official sources of information probably underestimate the true burden of disease attributable to listeria [4]. The Emerging Infections Programmes Network reported the incidence of listerial meningitis to be 0.05 cases per 100 000 population [5]; but the true incidence can be estimated to be higher [6].

We previously reported an increasing rate of unfavourable outcome among adults with listerial meningitis over a 14-year period, from 27% in 1998–2002 [7], [8], to 61% in 2006–2012 [9]. The emerging bacterial genotype sequence type (ST) 6 was identified as the main factor leading to this poorer prognosis [9]. Recently, *L. monocytogenes* clonal complex 6, comprising ST6, was associated with invasive disease, in particular meningitis, but data on outcome were not available [10]. *Listeria monocytogenes* ST6 isolates are typed as serotype B lineage II by classic serotyping methods. ST6 isolates are relatively infrequently cultured from contaminated food sources [10].

We investigated variation in the *L. monocytogenes* genome, in particular in the ST6 genotype, and its relation with clinical outcome in meningitis. We used whole bacterial genome sequencing of isolates from two nationwide cohort studies on listerial meningitis, and validated results in a nationwide surveillance study over a 30-year period.

## Materials and methods

### Nationwide clinical cohort

We identified adults as >16 years of age who had *L. monocytogenes* meningitis, as established by positive cerebrospinal fluid (CSF) culture, and were listed in the database of the Netherlands Reference Laboratory for Bacterial Meningitis between 1 October 1998 and 1 April 2002 [6], [7], and between 1 March 2006, and 1 April 2012 [9]. During these two periods, a prospective national cohort study ran in which patients with bacterial meningitis were included and their clinical characteristics were scored [9]. Patients or their legal representatives received written information concerning the study and were asked to give written informed consent for participation. Patients with hospital-acquired bacterial meningitis, neurosurgical procedures, or those within 1 month following neurosurgical procedure or neurotrauma were excluded. Patients with an altered immune status owing to splenectomy, diabetes mellitus, cancer, alcoholism, or the use of immunosuppressive drugs were considered immunocompromised, as were patients infected with human immunodeficiency virus. Neurological examination was performed at discharge, and outcome was scored according to the Glasgow Outcome Scale, with scores varying from 1 (death) to 5 (good recovery) [11]. A favourable outcome was defined as a score of 5, and an unfavourable outcome was defined as a score of 1–4. Adequate treatment was defined as amoxicillin 2 g six times per day or penicillin G 3 million units six times daily. If adequate treatment was not initiated on admission, number of days to adequate treatment was recorded. The studies were approved by the Medical Ethics Committee of the Academic Medical Centre, University of Amsterdam, the Netherlands.

### Bacterial whole genome sequencing

DNA from *L. monocytogenes* strains was extracted based on manufacturer’s protocol (Promega, Madison, WI, USA). Sequencing was performed using multiplexed libraries on the Illumina HiSeq platform to produce paired end reads of 100 nucleotides in length (Illumina, San Diego, CA, USA). Sequences of the bacterial samples were assembled *de novo* using SPAdes (version 3.6.0) with default parameters [12]. The median number of contigs was 11 (range 5–25), mean GC content 38%, average genome length 2 970 545 bp (range 2 859 080–3 105 945), and mean coverage 239-fold. Sequence types were determined from the whole genome sequences. PacBio sequencing was performed according to manufacturer protocols (Pacific Biosciences, Menlo Park, CA, USA).

### Data availability

Fastq sequences of bacterial isolates (accession numbers in see Supplementary material, Table S1) and nucleotide sequences of the bacteriophage (accession number Hx2000053476), plasmid pLMST6 (accession number Hx2000053471) and *emrC* gene (accession number Hx2000053480) have been deposited in the European Nucleotide Archive (ENA).

### Pan-genome generation and phylogenetic tree

Genome sequences were annotated with PROKKA, version 1.11 [13]. We used Roary (version 3.5.0) with default parameters to extract clusters of orthologous genes, referred to as gene groups, and create a core gene alignment at a sequence identity threshold of 95% [14]. This process identified a pan-genome of 6360 gene groups and a core genome (shared by 100% of strains) of 2177. The cumulative plot of the pan-genome suggested a closed pan-genome (see Supplementary material, Fig. S1).

A maximum likelihood phylogeny of single-nucleotide polymorphisms (SNPs) in the core genome of all sequenced isolates was produced with RAxML (version 7.8.6) assuming a general time reversible model of nucleotide substitution with a *γ*-distributed rate heterogeneity [15]. To generate the phylogenetic tree of the ST6 clade, isolates were mapped against an ST6 reference (accession number: NC_021829). For this analysis, the same parameter settings were used as for the phylogeny of all sequenced isolates.

Isolates from each of the four monophyletic groups were mapped to a reference in the same clade with SMALT (version 0.7.4, using default parameters), creating a pseudogenome alignment and phylogenetic tree using RAxML. This pseudogenome alignment and tree were used to infer areas of increased SNP density with Gubbins (version 1.7.4, default parameters) [16]. The algorithm converged to a stable phylogeny after four iterations.

### Bacterial genetic association study on clinical outcome

The sequences were mapped against an ST6 reference (accession number: NC_021829). The SNPs were called with bcftools (version 0.1.19) [17]. A matrix containing presence and absence of gene groups was generated from the Roary output [14]. As bacterial populations are generally clonal, standard methods for genome-wide analyses fail [18], GEMMA (version 0.94.1, default parameters) was used to perform a linear mixed model analysis for each SNP or gene group versus unfavourable outcome or mortality phenotype and determine p-values of association [19]. SNPs or gene groups present in <5% or >95% of isolates were excluded. The core SNP alignment was used as the design matrix of random effects to correct for population stratification through the generation of a centred relatedness matrix in GEMMA. No covariates were included. We considered a p-value (α of 0.05) corrected for multiple testing (166 839 tests) to be statistically significant (p <2.99 × 10^−7^). Bacteriophage sequences from isolates mapped to the NC_021829 reference isolate were extracted *in silico* by using the conserved gene boundaries of the *comK* gene as primers (see Supplementary material, Table S2). The percentage of sequence identity similarity was determined with BLAST [20]. A Fisher’s exact test was performed to assess the association of presence or absence of sequence elements with unfavourable outcome or mortality.

We used the core alignment of 20 ST6 and one single locus variant ST616 isolates and their sampling dates (range 1998–2011) to infer the date of emergence for the most recent common ancestor for isolates in the clonal expansion in this clade with a Bayesian analysis (BEAST, version 1.8.2) [21]. We used a strict clock model with an exponential growth population before, and ran Markov chain Monte Carlo for 75 million iterations. The first 7.5 million samples were discarded as burn-in.

### Nationwide surveillance study and functional validation

The Netherlands Reference Laboratory for Bacterial Meningitis receives 85%–90% of all bacterial meningitis isolates [22]. For this study, we included listerial isolates received by this laboratory between 1 January 1985 and 1 January 2015, irrespective of whether a cohort study on bacterial meningitis ran at that time and determined sequence type as described previously [23]. We allocated sequence types according to the multi-locus sequence type database at the Institut Pasteur (http://bigsdb.web.pasteur.fr/listeria/listeria.html). We performed a Mann–Whitney *U* test to assess whether the number of cases increased significantly for each 5-year time interval compared with the preceding time interval. A p-value <0.05 was considered statistically significant.

We assessed *L. monocytogenes* susceptibility to benzalkonium chloride by plating a loop of a bacterial suspension of approximately 10^8^ CFU/mL in phosphate-buffered saline on blood agar plates containing benzalkonium chloride in increasing concentration (0, 5, 10, 15, 20, 25, 30, 45 and 60 mg/L). Plates were evaluated after incubation over night at 37°C. Benzalkonium chloride tolerance was defined as growth inhibition at 60 mg/L. We determined the presence of quaternary ammonium efflux genes with PCR (see Supplementary material, Table S2). Susceptibility to amoxicillin and gentamicin as determined by MICs was assessed with E-test (BioMérieux, Marcy-l'Etoile, France) according to the manufacturer’s protocol. We tested for the association between *emrC* and benzalkonium chloride or antibiotic susceptibility with an ordinal logistic regression analysis, corrected for bacterial lineage, in the MASS package (version 7.3) in R (version 3.1.3). A p-value of <0.05 was considered statistically significant. Furthermore, the data were found to obey the proportional odds assumption using the Hmisc package (version 3.17; see Supplementary material, Table S3). Listerial strains were transformed by electroporation as previously described, with plasmid pLMST6, extracted from two ST6 isolates [24]. Minimum inhibitory concentrations for amoxicillin and gentamicin were assessed by E-test, as described above. Antibiotic susceptibility experiments after transformation were performed on regular blood agar plates and on plates supplemented with 30 mg/L benzalkonium chloride. All tests were performed blinded to presence of the plasmid in each isolate.

## Results

### Bacterial whole genome sequencing

**Fig. 1:**
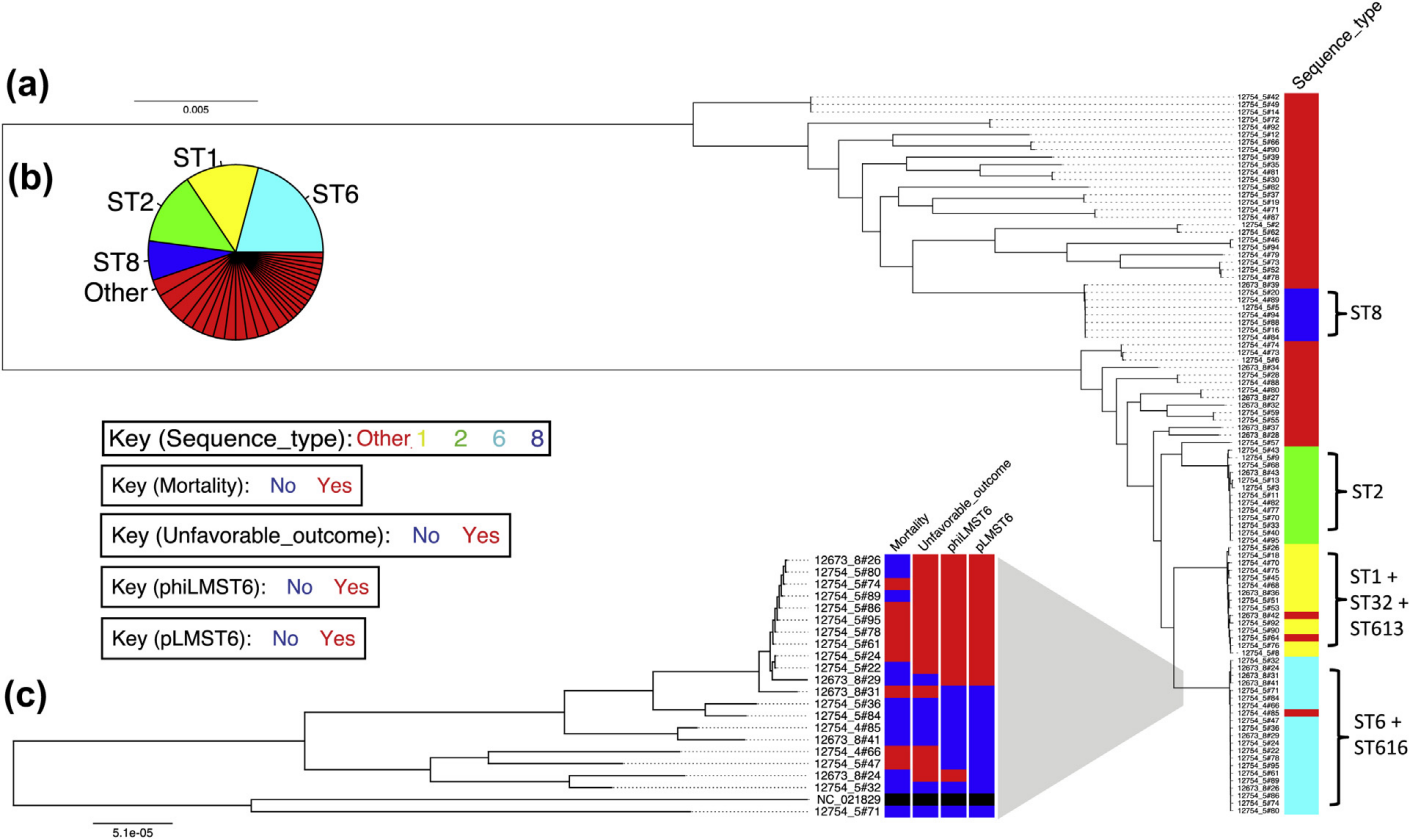
Phylogenetic tree of *Listeria monocytogenes* isolates causing meningitis and ST6 isolates specifically. (a) Maximum likelihood analysis of core single-nucleotide polymorphisms from 96 isolates shows a population divided in two lineages and four monophyletic groups. These monophyletic groups correspond to the largest sequence type (ST) groups in the collection and single locus variants of these groups. ST8 is coloured blue, ST6 is cyan, ST2 is green, ST1 is yellow, and other STs are in red. The mutation rate is represented on the horizontal axis. Distances on the vertical axis are for visualization purposes only. (b) Pie chart of STs of 96 isolates in the study cohort. Four major ST groups hold 55% of isolates. Remaining isolates are grouped with one or two isolates of similar ST or as singletons. (c) Maximum likelihood phylogenetic tree of ST6, ST616 (single locus variant to ST6) and ST6 reference (accession number: NC_021829), shows a clonal expansion within the monophyletic group. These isolates more often cause unfavourable outcome in patients compared with other ST6 isolates and carry the phiLMST6 phage and the pLMST6 plasmid. Mortality and unfavourable outcome for patients infected with these isolates, and presence of phiLMST6 and pLMST6 in each isolate is coloured red.

Bacterial whole genome sequencing was performed on 97 isolates; one isolate was excluded because of contamination. Genetic analysis of 96 isolates showed 33 different sequence types: ST6 in 20 (21%), ST1 in 13 (14%), ST2 in 13 (14%), ST8 in 7 (7%), and other sequence types in 43 (45%). The phylogenetic tree showed four distinct monophyletic groups branching from two major lineages (Fig. 1a). These are separated by 84 657 SNPs, indicating that about 3% of the genome was polymorphic. A comparative SNP density analysis showed recombination events in ST2, ST6 and ST8 monophyletic groups which were restricted to prophage insertions [16], so likely to be driven by phage population dynamics (see Supplementary material, Fig. S2).

### Nationwide clinical cohort

Eighty-five of 97 patients (88%) from whom the reference laboratory received a listerial CSF isolate in the study periods were included in the clinical cohort. Patients' characteristics are shown in Table 1. The case fatality rate was 31%, and 52% of the episodes had an unfavourable outcome. Sixty-four of 85 (75%) patients received adequate treatment at admission.

### Bacterial genetic association study on clinical outcome

**Fig. 2:**
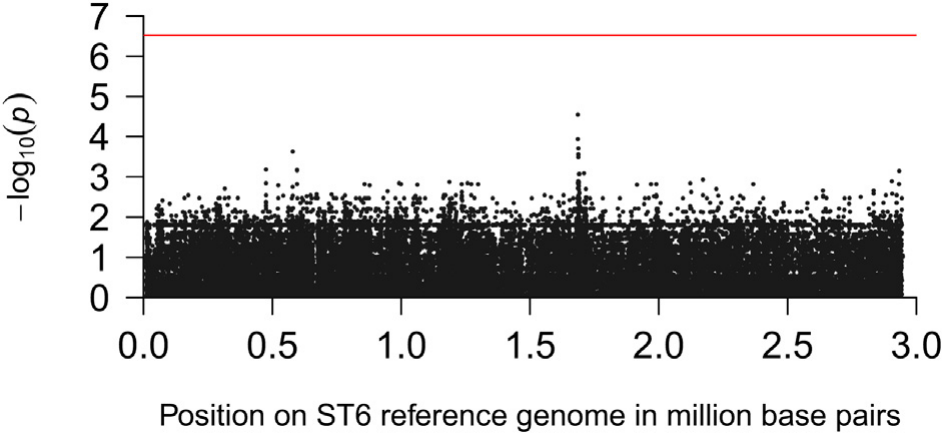
Manhattan plot for single-nucleotide polymorphisms (SNPs) associated with unfavourable outcome. Manhattan plot for SNPs associated with unfavourable outcome calculated by a univariate linear mixed model analysis. Sequences from 85 isolates were mapped against a ST6 reference and SNPs present in between 5% and 95% of isolates were called (*n* = 166 839). The −log_10_ of the p-value is plotted on the *y*-axis and the location of SNPs in the reference genome on the *x*-axis. The red horizontal line is the threshold for correction of multiple testing (Bonferroni correction). SNPs in a region around 1.65 Mbp on the reference genome have the lowest p-values. None of the SNPs reach statistically significant levels of association after correction for multiple testing.

There were 1952 gene groups and 166 839 SNPs present in between 5% and 95% of isolates. None of the gene groups or SNPs were significantly associated with unfavourable outcome or mortality after Bonferroni correction (Fig. 2; see Supplementary material, Figs. S3 and S4). However, because of statistical overcorrection for population stratification (see Supplementary material, Fig. S5) and extensive linkage disequilibrium in the listerial genome, Bonferroni correction is too strict [18]. The region around 1.65 million base pairs (Mbp) in the reference genome showed the strongest signal in the SNP association analysis with unfavourable outcome and was therefore explored further (Fig. 2). In this region in the reference genome we identified a prophage, which interrupts the master regulator for competence gene expression, *comK*, suggested to be a listerial virulence factor, which is not involved in DNA uptake [25]. The structural configuration of a prophage-interrupted *comK* gene was present in 22 of 85 *de novo* aligned clinical isolate sequences (26%) but was not associated with unfavourable outcome (Fisher’s exact test, p 0.62) or mortality (Fisher’s exact test, p 0.43) in our cohort. However, another prophage with high homology to the *comK*-interrupting phage (median sequence identity, 66%; range, 65%–70%) was not present in the reference isolate and identified in 12 clinical isolates and drove the observed association with unfavourable outcome. This novel phage (phiLMST6; accession number Hx2000053476) was present in ST6 isolates only and in the phylogenetic tree of ST6, 11 of 12 (92%) isolates carrying phiLMST6 were clustered and formed a clonal expansion (Fig. 1c). These isolates were associated with unfavourable outcome (occurring in 11 of 12 patients infected with isolates carrying phiLMST6 (92%) versus 33 of 73 patients infected with isolates without phiLMST6 (45%); Fisher’s exact test, p 0.004) but not mortality (6 of 12 (50%) versus 20 of 53 (27%); Fisher’s exact test, p 0.17). The phiLMST6 phage consisted of 66 genes, of which 47/66 (71%) encode hypothetical proteins, 14/66 (21%) encode phage structural proteins, 2/66 (2%) are recombinases and 3/66 (5%) encode annotated genes: a methylase, an amidase and a DNA-binding protein (see Supplementary material, Fig. S6).

### A novel plasmid containing a quaternary ammonium efflux pump

Upon manually characterizing the mobile genetic elements in the ST6 sequences, phiLMST6 was found to co-occur with a novel plasmid (Fisher’s exact test, p <0.001) that was present in the same subset of ST6 isolates (*n* = 11) forming the clonal expansion (Fig. 1c) and one ST9 isolate (see Supplementary material, Fig. S7). It was absent from the reference isolate and therefore not included in the association analysis. The plasmid, named pLMST6, is 4378 bp in size and consists of seven open reading frames larger than 150 bp, encoding a putative Tet/AcR-like transcriptional regulator, a recombination–mobilization protein, a plasmid replicase, a CopG like transcriptional regulator, two hypothetical proteins and a quaternary ammonium efflux protein homologous to a multidrug efflux protein Emr, named EmrC (gene: *emrC*; sequence homologous to publicly available sequence WP_061092474.1, see Supplementary material, Fig. S8). A closed sequence of pLMST6 was reproduced using long-read-length sequencing techniques (PacBio; accession number Hx2000053471). This plasmid had no sequence homology with the previously discovered quaternary ammonium compound tolerance plasmid pLM80 [26] and the *emrC* gene had partial homology to the quaternary ammonium efflux gene on the Tn*6188* transposon (query coverage 84%, identity 73% by discontiguous megablast) [27]. One of the most widely used quaternary ammonium compounds is benzalkonium chloride, which is extensively used in the food-processing industry as a disinfectant and sterilization agent [28]. To test for a temporal correlation with the emergence of pLMST6 containing *emrC* in our collection, a Bayesian dating analysis was performed [21]. The inferred date for emergence of the most recent common ancestor for ST6 isolates carrying pLMST6 was 15 years (95% highest posterior density interval, 8–32 years; see Supplementary material, Fig. S9).

Quaternary ammonium efflux proteins have broad substrate specificity. We therefore then tested the hypothesis that pLMST6 results in decreased antibiotic susceptibility in isolates from a nationwide cohort.

### Nationwide surveillance study and functional validation

**Fig. 3:**
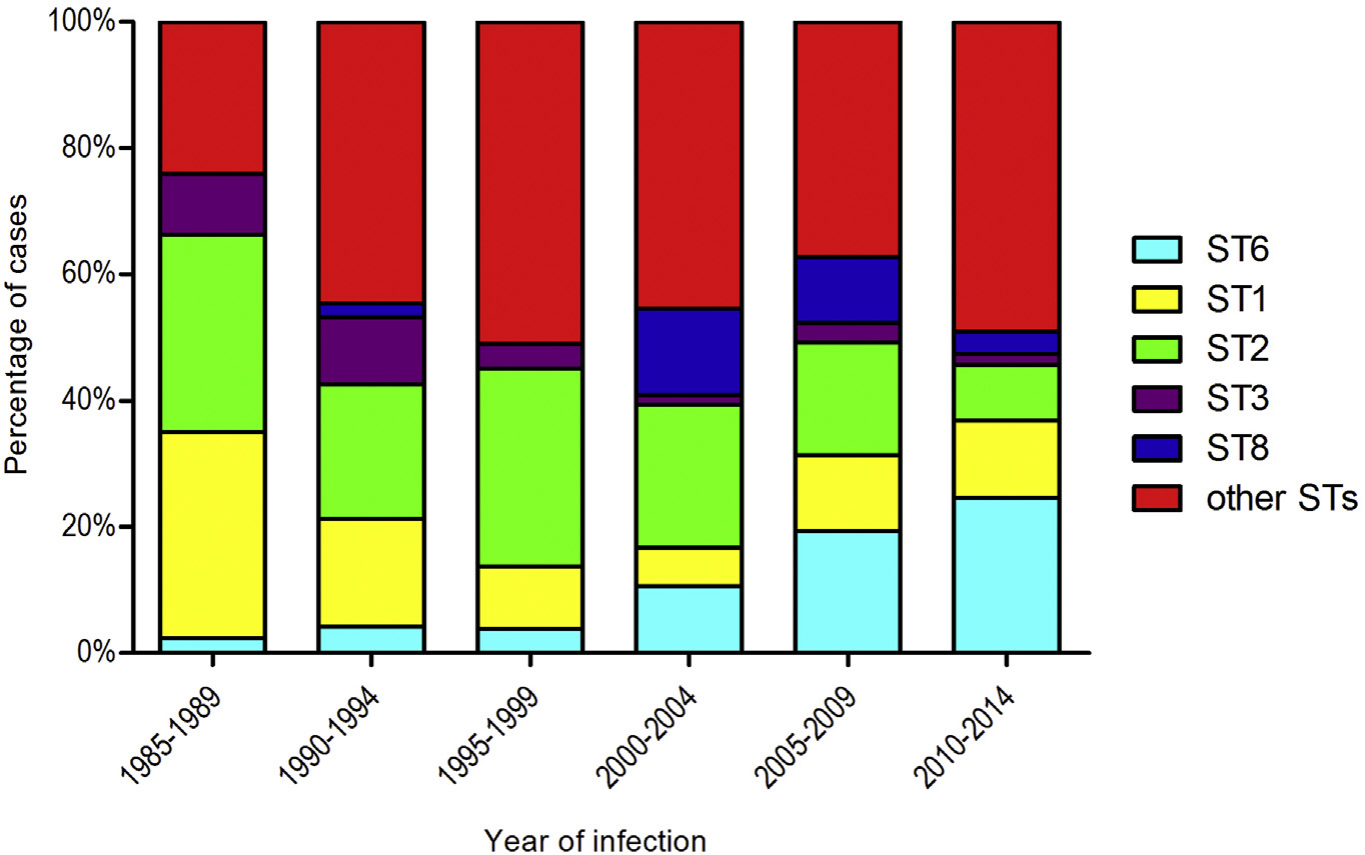
Absolute number of *Listeria monocytogenes* meningitis cases for the largest multi locus sequence type groups from 1985 to 2014 in The Netherlands. The absolute number of cases per listerial multi-locus sequence type causing meningitis in the Netherlands is shown for six time intervals. The number of listeria meningitis cases did not vary significantly over the time intervals. Data for the five most common sequence types (STs) is shown. Remaining STs are clustered in the category ‘other STs’. In the time interval 1985–1989, ST1 and ST2 were the dominant STs. In the following years, ST1 and ST2 cases decreased whereas ST6 cases increased. In 2010–2014, ST6 caused disease in most cases, followed by ST1 and ST2. The number of ST6 cases increased significantly over the years (Mann–Whitney *U* test, p <0.001). ST6 is coloured cyan, ST1 is yellow, ST2 is green, ST3 is purple, ST8 is blue, and other STs are red.

The reference laboratory received 1084 listerial isolates between 1 January 1985 and 1 January 2015; 371 of these isolates were from CSF, the remaining 713 were isolated from blood. For this study, we analysed all 371 CSF isolates and a randomly selected set of 74 blood isolates. Multi-locus sequence typing showed 62 different sequence types; most common were ST2 (in 102 isolates; 23%), ST1 (68; 15%), and ST6 (44; 10%). The proportion of ST6 cases increased significantly over the years (Fig. 3; Mann–Whitney *U* test, p <0.001), and the 5-year interval of ST6 emergence in this data set was in line with the inferred date from Bayesian dating for emergence of the most recent common ancestor to isolates carrying pLMST6 containing *emrC*.

**Fig. 4:**
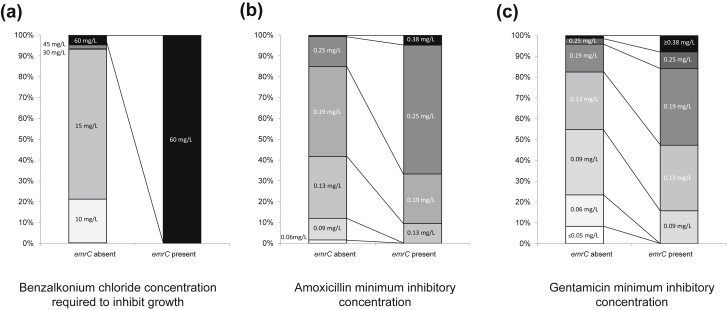
Proportion of isolates with or without the *emrC* gene with benzalkonium chloride tolerance and MIC to antibiotics. Proportion of isolates with or without the *emrC* gene with benzalkonium chloride tolerance and MICs to amoxicillin and gentamicin. A collection of 445 isolates were tested. Values of p for association were determined with an ordinal logistic regression analysis, corrected for bacterial lineage. (a) Isolates with or without *emrC* versus benzalkonium chloride concentration (p <0.001). (b) Isolates with or without *emrC* versus amoxicillin MICs (p <0.001). (c) Isolates with or without *emrC* versus gentamicin MICs (p 0.003).

The *emrC* gene was detected in 21 of the 445 isolates (5%). The distribution of sequence types containing the gene was: ST6 in 17 (81%) isolates, and one (5%) in ST9, ST8, ST101 and ST576. Presence of the quaternary ammonium efflux gene was associated with benzalkonium chloride tolerance (median 60 mg/L, range 60–60 versus 15 mg/L, range 5–60; ordinal logistic regression p <0.001) and increased MICs for gentamicin (median 0.125 mg/L, range 0.094–0.5 versus 0.094 mg/L, range 0.023–1.5; ordinal logistic regression p 0.003) and amoxicillin (median 0.25 mg/L, range 0.125–0.38 versus 0.19 mg/L, range 0.032–0.38; ordinal logistic regression p <0.001) (Fig. 4).

To further investigate whether pLMST6 caused benzalkonium chloride tolerance and decreased antibiotic susceptibility we transformed pLMST6 into three strains naive for this plasmid (two ST6 and one ST29). After transformation, growth was inhibited at benzalkonium chloride concentrations of 60 mg/L, compared with 15 mg/L before transformation, for all three recipient strains. For gentamicin, MICs increased from 0.125 to 0.19 mg/L, 0.19 to 0.38 mg/L, and 0.094 to 0.19 mg/L on blood agar not supplemented with benzalkonium chloride, and from 0.125 to 1.0 mg/L, 0.19 to 1.0 mg/L, and 0.094 to 1.0 mg/L in medium supplemented with benzalkonium chloride, suggesting plasmid induction or increased gene expression [29]. For amoxicillin, MICs before and after transformation were similar on blood agar with or without benzalkonium chloride (see Supplementary material, Table S4).

## Discussion

We identified a novel listerial plasmid and an efflux transporter, *emrC*, to be associated with the emergence of meningitis caused by *L. monocytogenes* ST6 in the Netherlands, possibly through decreased susceptibility to disinfecting agents. Infection with bacteria carrying the plasmid and efflux transporter was associated with unfavourable disease outcome in patients. Whether this is caused by *emrC* induced antibiotic treatment failure, a different plasmid gene increases virulence or the phiLMST6 bacteriophage modulates virulence, remains to be determined. The *emrC* gene encodes an efflux protein that pumps quaternary ammonium compounds out of the cell and increases the capacity to form a biofilm [30], resulting in benzalkonium chloride tolerance. Benzalkonium chloride is extensively used in the food-processing industry as a disinfectant and sterilization agent [28]. Our findings might imply that disinfectants used in the food-processing industry select for resistance mechanisms that may, inadvertently, lead to an increase in disease severity and poor outcome.

A limitation of this study is that we were unable to generate *emrC* plasmid knock-outs to pinpoint *emrC* as the causal factor to explain the observed benzalkonium chloride tolerance phenotype. However, given the currently known functions of the annotated genes, this is the most convincing explanation. Because of lack of a sufficiently sensitive animal model of listerial meningitis, we were unable to compare pLMST6 naive and pLMST6 transformed isolates to directly assess virulence.

We show an association between isolates carrying the plasmid and decreased antibiotic susceptibility. However, it remains to be determined whether this is caused by *emrC*-mediated antibiotic efflux, efflux of other antibacterial compounds, increased capacity to form a biofilm or whether this is due to increased bacterial membrane stability.

Genes conferring decreased susceptibility to benzalkonium chloride have been linked to listerial virulence in laboratory studies [31]. These studies showed that genes conferring tolerance to disinfecting agents used in the food-processing industry, including benzalkonium chloride, are strongly associated with resistance to antimicrobials, such as gentamicin and ciprofloxacin [32], consistent with our findings. Listerial isolates resistant to benzalkonium chloride showed cross-resistance to several environmental stress factors in a laboratory setting, for example heat, osmotic and oxidative stresses [33]. Quaternary ammonium efflux pumps have also been described in other bacterial species, such as *Staphylococcus aureus*, *Pseudomonas aeruginosa* and the food-borne pathogen *Camplyobacter* spp. [31], [34], [35]. We are the first to show a clinically relevant association of this long-feared resistance mechanism [36].

We observed an increase of listerial meningitis caused by ST6 over a 30-year period in the Netherlands. Common sequence types were ST6, ST1, ST2 and ST8. This is comparable to the rank order of major sequence types described in a recent study from France [10]. In this study, 83 of 628 listerial isolates (13%) causing central nervous system disease belonged to clonal complex 6. Isolates were collected between 2005 and 2013, but no temporal trends were provided, precluding statements about emergence of ST6 in France. A sequencing study from Australia including 225 invasive listerial isolates, described no ST6 isolates in the major clades [37]. A global sampling study including 300 disease and environmental listerial isolates from five continents showed clonal complex 6 to be present mainly in Europe and North America [38]. A recent analysis of two *L. monocytogenes* outbreaks in Denmark showed ST6 to be responsible for one of the outbreaks, confirming this sequence type to be involved in disease in other European countries [39]. Additional surveillance studies are needed to determine whether clonal expansion or acquisition of *emrC* harbouring listerial isolates causing severe invasive disease must be regarded as a global phenomenon.

Our study stresses the need for routine surveillance studies on bacterial genetics and clinical outcome to monitor the geographical distribution and potential emergence of resistant listerial phenotypes. To avoid the selection of *L. monocytogenes* harbouring *emrC* that are associated with unfavourable outcome, possibly through therapeutic failures, strict regulation of disinfectant procedures in food-processing plants might be warranted.

## Transparency declaration

The authors declare no conflicts of interest.

## Figures and Tables

**Table 1:** Baseline table of patient characteristics for 85 patients

Characteristic	Value
Median age (range), years	68 (20–95)
Male sex, *n*/*N* (%)	52/85 (61)
Predisposing conditions, *n*/*N* (%)	
Immunocompromised	60/85 (71)
Score on Glasgow Coma Scale at admission, *n*/*N* (%)^a^	
<14 (indicating change in mental status)	53/85 (62)
≤8 (indicating coma)	7/85 (8)
Focal neurological deficits, *n*/*N* (%)	31/85 (36)
Score on the Glasgow Outcome Scale, *n*/*N* (%)^b^	
1 (death)	26/85 (31)
2 (vegetative state)	0/85 (0)
3 (severe disability)	6/85 (7)
4 (moderate disability)	12/85 (14)
5 (mild or no disability)	41/85 (48)
Unfavourable outcome, *n*/*N* (%)	44/85 (52)
Adequate treatment at admission, *n*/*N* (%)	64/85 (75)

a Scores on the Glasgow Coma Scale can range from 3 to 15, with 15 indicating a normal level of consciousness.

b Scores on the Glasgow Outcome Scale can range from 1 to 5, with 5 indicating mild or no disability (the patient is able to return to work or school).

## Appendix A Supplementary material

The following supplementary materials are available for this article:

**Fig. S1.** *Listeria monocytogenes* pan-genome.

**Fig. S2.** Recombination in the four largest clades of the phylogeny.

**Fig. S3.** Single-nucleotide polymorphisms associated with mortality.

**Fig. S4.** Gene groups associated with unfavourable outcome and mortality.

**Fig. S5.** Quantile–quantile plot for single-nucleotide polymorphisms versus unfavourable outcome.

**Fig. S6.** Majority of genes of the phage phiLMST6 are structural phage genes and genes coding for proteins without known functions.

**Fig. S7.** Plasmid pLMST6 in isolates distributed over the phylogenetic tree of *Listeria monocytogenes*.

**Fig. S8.** Plasmid pLMST6 containing seven open reading frames, one of which encodes a quaternary ammonium efflux protein, EmrC.

**Fig. S9.** Inferred date from Bayesian dating analysis for the emergence of the most recent common ancestor to pLMST6 harbouring ST6 isolates.

**Table S1.** EMBL-ENA accession numbers for the fastq files of the whole genome sequences

**Table S2.** Primer sequences used in this manuscript

**Table S3.** Proportional odds assumptions for the ordinal logistic regression analysis assessing the association of quaternary ammonium tolerance (qac) genes with amoxicillin and gentamicin E-test values and benzalkonium chloride tolerance

**Table S4.** Transformation of pLMST6 into naïve strains leads to decreased benzalkonium chloride susceptibility and increased E-test values for gentamicin but not amoxicillin
