# Supplementary material for: Benzalkonium tolerance genes and outcome in Listeria monocytogenes meningitis
Source: Clin Microbiol Infect. 2017 Apr;23(4):265.e1–7. doi: 10.1016/j.cmi.2016.12.008 (PMC5392494; doi:10.1016/j.cmi.2016.12.008)
Supplement: Supplementary file 1 [file mmc1.doc]

**Supplementary Figures and Tables**

**Table of contents**

Supplementary Figure S1....................................................................................................... 2

Supplementary Figure S2....................................................................................................... 3

Supplementary Figure S3....................................................................................................... 4

Supplementary Figure S4....................................................................................................... 5

Supplementary Figure S5....................................................................................................... 6

Supplementary Figure S6....................................................................................................... 7

Supplementary Figure S7....................................................................................................... 8

Supplementary Figure S8....................................................................................................... 9

Supplementary Figure S9....................................................................................................... 10

Supplementary Table S1......................................................................................................... 12

Supplementary Table S2......................................................................................................... 13

Supplementary Table S3......................................................................................................... 15

Supplementary Table S4......................................................................................................... 16

**Figure S1.** *Listeria monocytogenes* pan-genome.

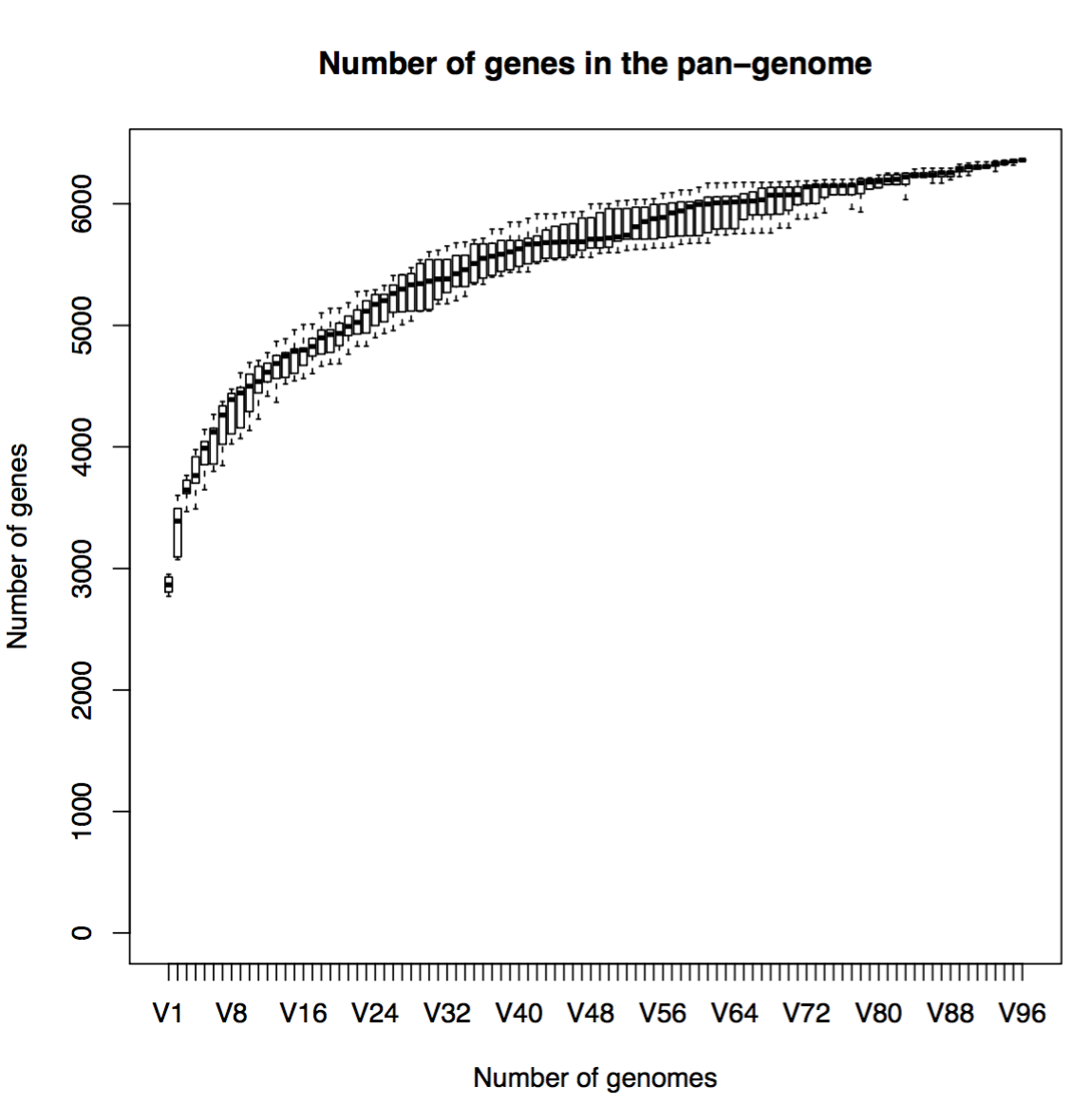
**Footnote** **Supplementary Figure 1.**

The cumulative number of clusters of orthologous genes (COGs), or gene groups, is plotted, with margins representing varying cumulative sequences for genomes in the collection. The curve is linear on a log-transformed scale, suggesting a closed pan-genome.

**Figure S2.** Recombination in the four largest clades of the phylogeny.


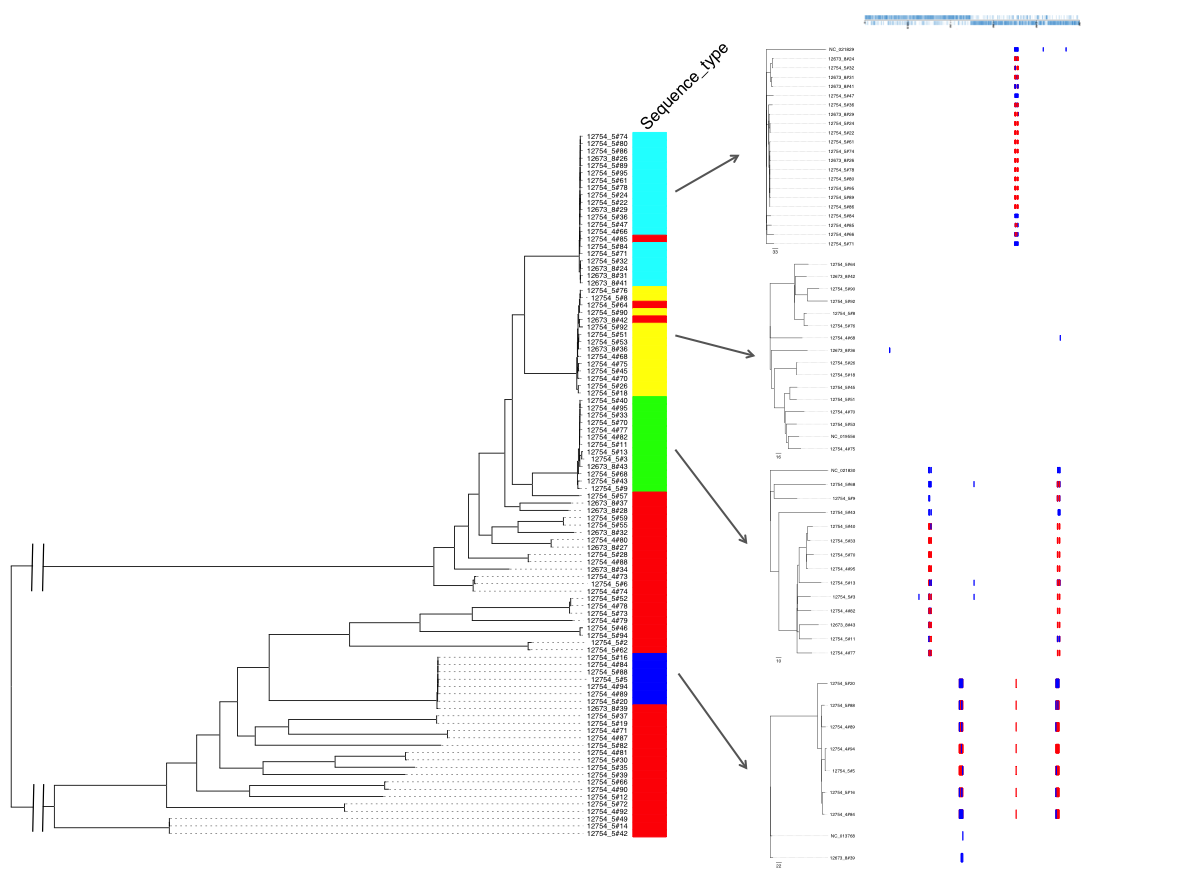


**Footnote Supplementary Figure 2.**

A single nucleotide polymorphism (SNP) density analysis was performed in the four largest monophyletic groups, corresponding to the sequence type groups ST6, ST1, ST2 and ST8 and single locus variants in those groups. In ST6, ST2 and ST8 SNP dense regions were discovered corresponding to phage insertions in the genomes of isolates in these monophyletic groups. SNP dense regions are represented in the plot as blue and red blocks, depending on whether the SNP dense region (block) is unique to one isolate (blue) or shared by multiple isolates through common descent (red). The horizontal position of the blocks represents their position in the alignment. After removing these areas of recombination the phylogeny for each monophyletic group was redrawn based on sporadic mutations only.

**Figure S3.** SNPs associated with mortality.


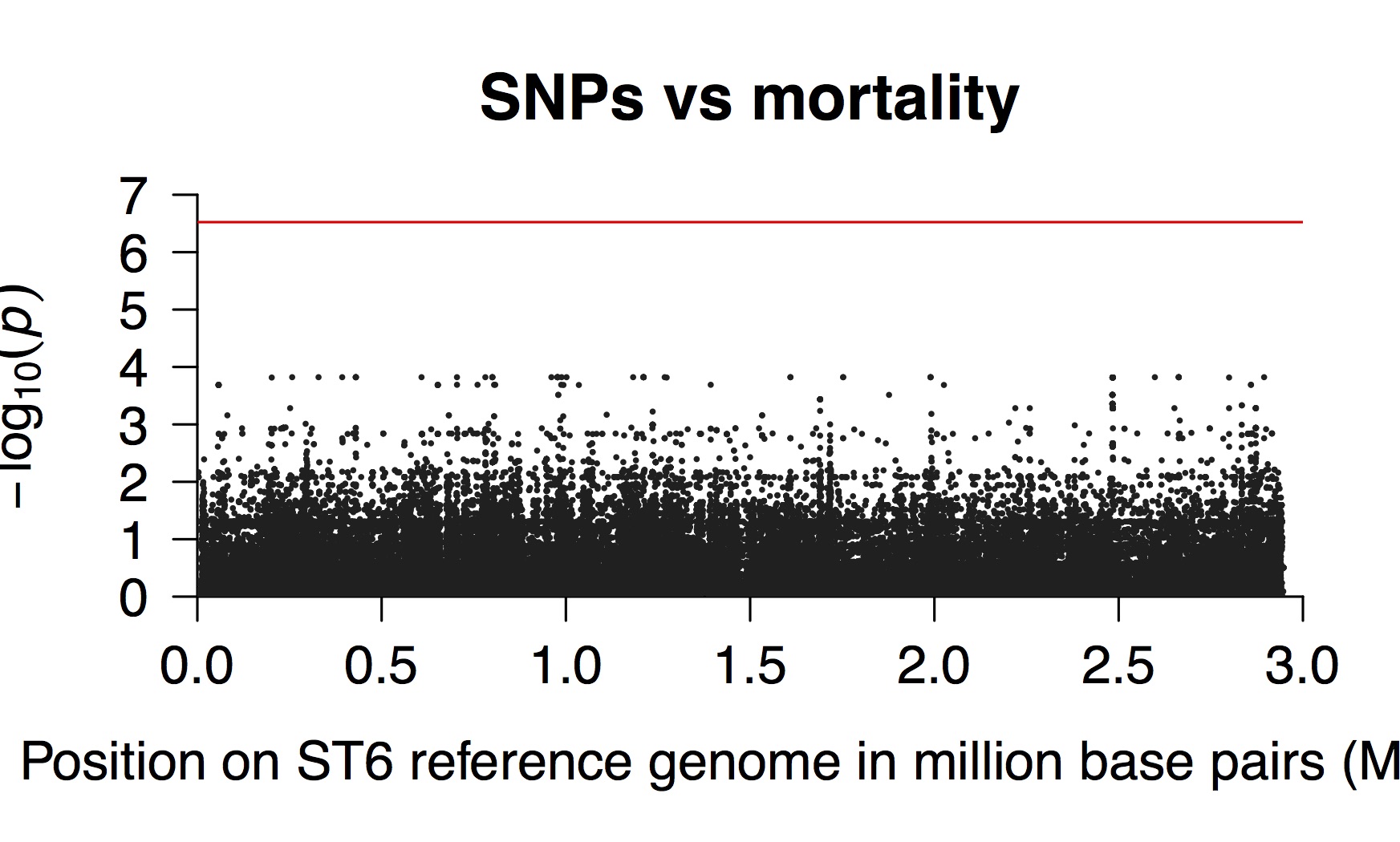


**Footnote Supplementary Figure 3.**

Manhattan plot for SNPs associated with mortality calculated by a univariate linear mixed model analysis. SNPs were mapped to a ST6 reference and present in less than 95% and more than 5% of isolates. On the y-axis the minus 10 log of the p-value, on the x-axis the location of SNPs in the reference genome. The red horizontal line is the threshold for correction of multiple testing (Bonferroni correction). None of the SNPs reach statistically significant levels of association after correction for multiple testing.

**Figure S4.** Gene groups associated with unfavourable outcome and mortality.

**Footnote Supplementary Figure 4.**

Univariate linear mixed model analysis for gene groups present in less than 95% and more than 5% of isolates. On the y-axis the minus 10 log of the p-value, on the x-axis the occurrence of gene groups in isolates from the collection from most (left) to least (right). The red horizontal line is the threshold for correction of multiple testing (Bonferroni correction). The regions to the left of the plot, with similar p-values, correspond to gene groups unique to lineage I or lineage II isolates only. A. Gene groups versus unfavourable outcome. B. Gene groups versus mortality. None of the gene groups reach statistically significant levels of association after correction for multiple testing.

**Figure S5.** Quantile-quantile plot for SNPs versus unfavourable outcome.


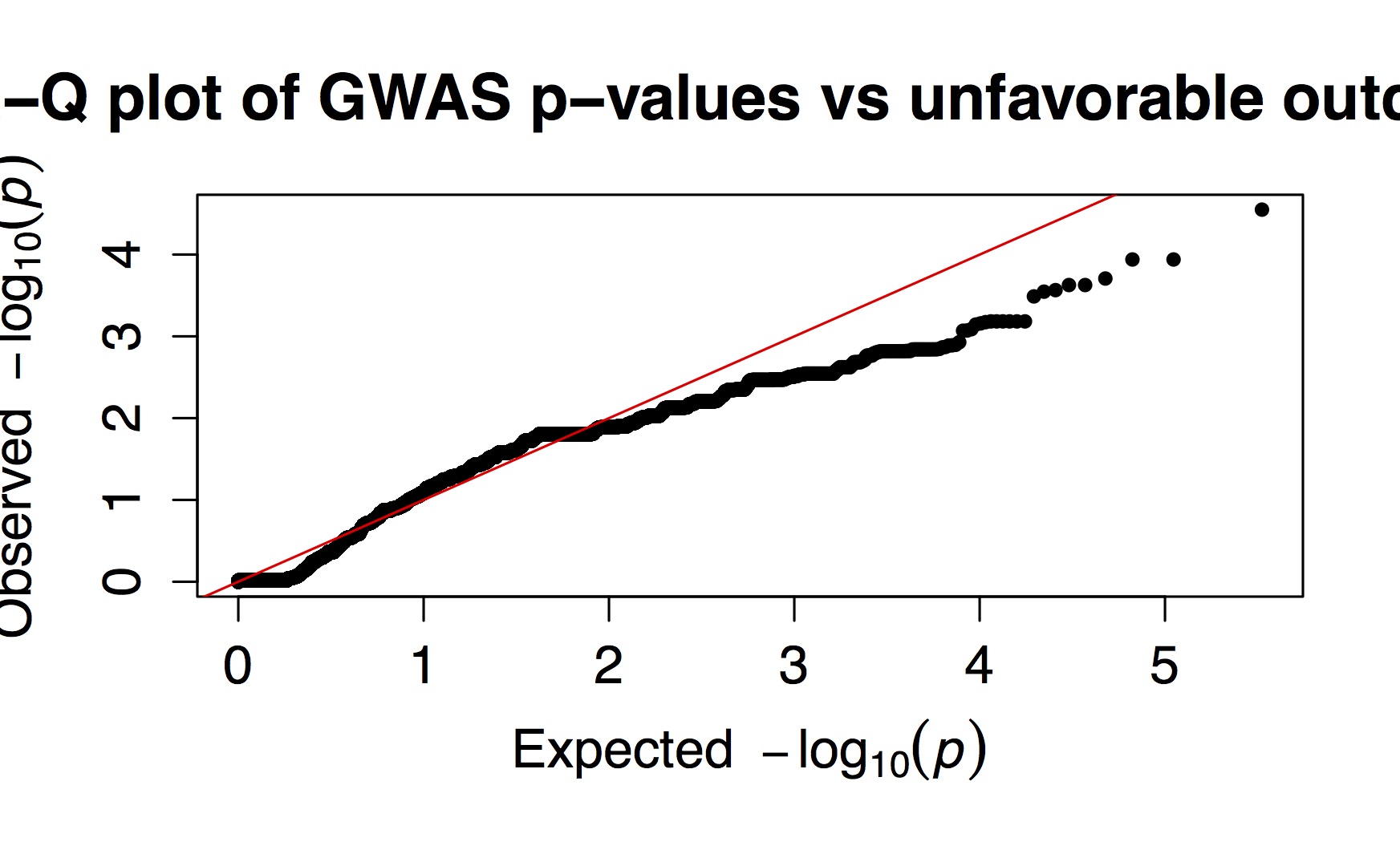


**Footnote Supplementary Figure 5.**

The quantile-quantile plot for p-values was determined in a linear mixed model and shows the expected versus observed p-values for SNP against unfavourable outcome. Observed p-values for unfavourable outcome are less than expected by chance (deviation from red line to the right). This can be caused by overcorrection for population structure, a known limitation of the linear mixed model.

**Figure S6.** Majority of genes of the phage phiLMST6 are structural phage genes and genes coding for proteins without known functions.


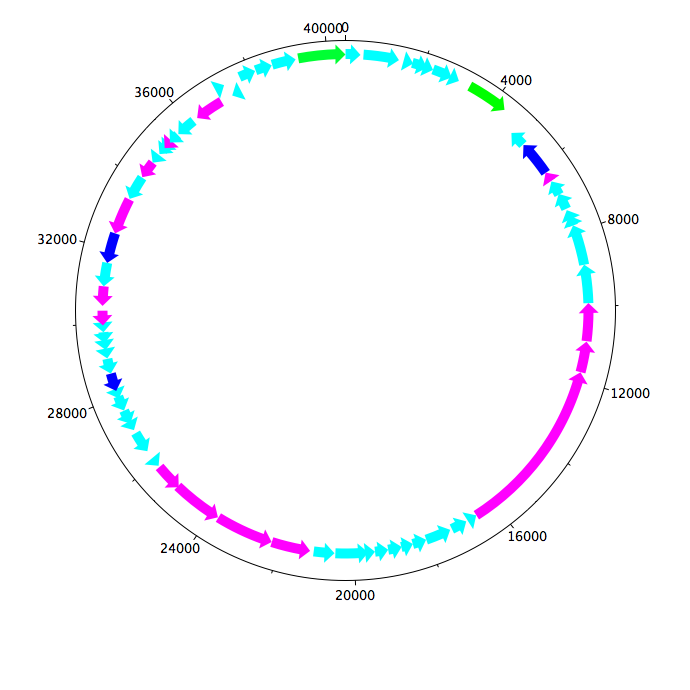


**Footnote Supplementary Figure 6.**

This Figure shows the 66 genes in the phage phiLMST6. Annotated genes are coloured blue and purple. Genes coding for phage structural proteins are shown in purple (14 genes), genes coding for proteins with other functions are shown in blue (3 genes). Genes coding for hypothetical proteins or proteins with unknown function are in cyan (47 genes). Recombinases are shown in green (2 genes).

**Figure S7.** Plasmid pLMST6 in isolates distributed over the phylogenetic tree of *L. monocytogenes*.

**Footnote Supplementary Figure 7.**

The phylogenetic tree of sequenced listeria isolates is shown. The sequence type is shown in the first column. Presence or absence of the novel plasmid pLMST6 is plotted in the second. pLMST6 is present in a subset of ST6 isolates and one ST9 isolate.

**Figure S8.** Plasmid pLMST6 containing 7 open reading frames, one of which encodes a quaternary ammonium efflux protein, EmrC.

**Footnote Supplementary Figure 8.**

The novel plasmid pLMST6 is 4378 bp in size and consisted of 7 open reading frames larger than 150 bp, encoding a putative Tet/AcR like transcriptional regulator, a putative recombination-mobilization protein, a putative plasmid replicase, a putative CopG like transcriptional regulator, two hypothetical proteins and a quaternary ammonium efflux protein, EmrC.

**Figure S9.** Inferred date from Bayesian dating analysis for the emergence of the most recent common ancestor to pLMST6 harbouring ST6 isolates.

**Footnote Supplementary Figure 9.**

Correlation between sequence variation of ST6 isolates and their sampling dates. Plotted on the y-axis is the time in years for divergence of different isolates. The inferred date for emergence of the most recent common ancestor to ST6 isolates carrying the novel pLMST6 plasmid was 15 years (95% highest posterior density interval, 8–32 years).

**Table S1. EMBL-ENA accession numbers for the fastq files of the whole genome sequences.**

| **Lane_ID** | **ERR** | **Lane_ID** | **ERR** |
| --- | --- | --- | --- |
| 12673_8#24 | ERR555023 | 12754_5#28 | ERR564066 |
| 12673_8#26 | ERR555025 | 12754_5#3 | ERR564041 |
| 12673_8#27 | ERR555026 | 12754_5#30 | ERR564068 |
| 12673_8#28 | ERR555027 | 12754_5#32 | ERR564070 |
| 12673_8#29 | ERR555028 | 12754_5#33 | ERR564071 |
| 12673_8#31 | ERR555030 | 12754_5#35 | ERR564073 |
| 12673_8#32 | ERR555031 | 12754_5#36 | ERR564074 |
| 12673_8#34 | ERR555033 | 12754_5#37 | ERR564075 |
| 12673_8#36 | ERR555035 | 12754_5#39 | ERR564077 |
| 12673_8#37 | ERR555036 | 12754_5#40 | ERR564078 |
| 12673_8#39 | ERR555038 | 12754_5#42 | ERR564080 |
| 12673_8#41 | ERR555040 | 12754_5#43 | ERR564081 |
| 12673_8#42 | ERR555041 | 12754_5#45 | ERR564083 |
| 12673_8#43 | ERR555042 | 12754_5#46 | ERR564084 |
| 12754_4#66 | ERR564009 | 12754_5#47 | ERR564085 |
| 12754_4#68 | ERR564011 | 12754_5#49 | ERR564087 |
| 12754_4#70 | ERR564013 | 12754_5#5 | ERR564043 |
| 12754_4#71 | ERR564014 | 12754_5#51 | ERR564089 |
| 12754_4#73 | ERR564016 | 12754_5#52 | ERR564090 |
| 12754_4#74 | ERR564017 | 12754_5#53 | ERR564091 |
| 12754_4#75 | ERR564018 | 12754_5#55 | ERR564093 |
| 12754_4#77 | ERR564020 | 12754_5#57 | ERR564095 |
| 12754_4#78 | ERR564021 | 12754_5#59 | ERR564097 |
| 12754_4#79 | ERR564022 | 12754_5#6 | ERR564044 |
| 12754_4#80 | ERR564023 | 12754_5#61 | ERR564099 |
| 12754_4#81 | ERR564024 | 12754_5#62 | ERR564100 |
| 12754_4#82 | ERR564025 | 12754_5#64 | ERR564102 |
| 12754_4#84 | ERR564027 | 12754_5#66 | ERR564104 |
| 12754_4#85 | ERR564028 | 12754_5#68 | ERR564106 |
| 12754_4#87 | ERR564030 | 12754_5#70 | ERR564108 |
| 12754_4#88 | ERR564031 | 12754_5#71 | ERR564109 |
| 12754_4#89 | ERR564032 | 12754_5#72 | ERR564110 |
| 12754_4#90 | ERR564033 | 12754_5#73 | ERR564111 |
| 12754_4#92 | ERR564035 | 12754_5#74 | ERR564112 |
| 12754_4#94 | ERR564037 | 12754_5#76 | ERR564114 |
| 12754_4#95 | ERR564038 | 12754_5#78 | ERR564116 |
| 12754_5#11 | ERR564049 | 12754_5#8 | ERR564046 |
| 12754_5#12 | ERR564050 | 12754_5#80 | ERR564118 |
| 12754_5#13 | ERR564051 | 12754_5#82 | ERR564120 |
| 12754_5#14 | ERR564052 | 12754_5#84 | ERR564122 |
| 12754_5#16 | ERR564054 | 12754_5#86 | ERR564124 |
| 12754_5#18 | ERR564056 | 12754_5#88 | ERR564126 |
| 12754_5#19 | ERR564057 | 12754_5#89 | ERR564127 |
| **Lane_ID**  12754_5#2 | **ERR**  ERR564040 | **Lane_ID**  12754_5#9 | **ERR**  ERR564047 |
| 12754_5#20 | ERR564058 | 12754_5#90 | ERR564128 |
| 12754_5#22 | ERR564060 | 12754_5#92 | ERR564130 |
| 12754_5#24 | ERR564062 | 12754_5#94 | ERR564132 |
| 12754_5#26 | ERR564064 | 12754_5#95 | ERR564133 |

**Table S2.** Primer sequences used in this manuscript.

A. Primers for the *comK* gene boundaries in sequences mapped to ST8 references.

Forward primer:

TACTAGAGCCATTCAATAGTAACTTGTTCACCGTCGATATAAATTTTATTAATTA

Reverse primer:

CTAATTATTAACAATAGATTGATGTTGTGTATACTCGTCGTAGATAGTCCTTAAA

B. Primers for the *emrC* gene.

Forward primer:

CGCCTATGCCATTTGGTCTG

Reverse primer:

TACTTGCTTCACCGTGCCC

**Footnote Supplementary Table 2.**

A. Nucleotide sequences of the *in silico* primers targeting the *comK* gene boundaries. Various different bacteriophages from multiple clinical isolates map to the *comK* interrupting bacteriophage in the reference sequence for the ST8 reference NC_013768. These sequences were extracted by an *in silico* PCR method guided by these primers. B. Nucleotide sequences of the primers used to target the *emrC* gene. These primers were used in an *in vitro* PCR assay to test for presence of this gene in 445 listerial isolates.

**Table S3.** Proportional odds assumptions for the ordinal logistic regression analysis assessing the association of quaternary ammonium tolerance (qac) genes with amoxicillin and gentamicin E-test values and benzalkonium chloride tolerance.

as.numeric(Amoxy) N=445

+-------+----+-------+---------+

| | |Y>=0.19|Y>=0.25 |

+-------+----+-------+---------+

|Qac |none|0 |-2.060460|

| |qac | 0 |-1.558145|

+-------+----+-------+---------+

|Overall| |0 |-1.944764|

+-------+----+-------+---------+

+-------+----+-------+---------+

| | |Y>=0.25|Y>=0.38 |

+-------+----+-------+---------+

|Qac |none|0 |-3.216800|

| |qac | 0 |-3.688879|

+-------+----+-------+---------+

|Overall| |0 |-3.154097|

+-------+----+-------+---------+

as.numeric(Genta) N=445

+-------+----+--------+---------+

| | |Y>=0.125|Y>=0.19 |

+-------+----+--------+---------+

|Qac |none|0 |-1.348342|

| |qac | 0 |-3.167583|

+-------+----+--------+---------+

|Overall| |0 |-1.399242|

+-------+----+--------+---------+

+-------+----+-------+---------+

| | |Y>=0.19|Y>=0.25 |

+-------+----+-------+---------+

|Qac |none|0 |-1.578398|

| |qac | 0 |-1.335001|

+-------+----+-------+---------+

|Overall| | 0 |-1.553652|

+-------+----+-------+---------+

+-------+----+-------+----------+

| | |Y>=0.25|Y>=0.38 |

+-------+----+-------+----------+

|Qac |none|0 |-1.1277406|

| |qac | 0 |-0.7444405|

+-------+----+-------+----------+

|Overall| |0 |-1.0799519|

+-------+----+-------+----------+

**Footnote Supplementary Table 3.**

The linear predicted values from the regressed dependent value (gentamicin and amoxicillin E-test values) on our predictor variable (qac or none) are shown for the categories of dependent variable with the highest numbers, without the parallel slopes assumption. These values were calculated with the Hmisc package in R. Most of these values are comparable, therefore the parallel slopes assumption holds.

**Table S4.** Transformation of pLMST6 into naïve strains leads to decreased benzalkonium chloride susceptibility and increased E-test values for gentamicin but not amoxicillin.

| **Donor strain** | **Growth medium** | **Sequence type** | **Growth inhibition at BZK concentration (mg/L)** | **Gentamicin MIC (mg/L)** | **Amoxicillin MIC (mg/L)** |
| --- | --- | --- | --- | --- | --- |
| 1 | regular | 6 | 60 | 0.125 | 0.19 |
| 1 | BZK suppl.* | 6 | - | 0.5 | 0.094 |
| 2 | regular | 6 | 60 | 0.125 | 0.19 |
| 2 | BZK suppl. | 6 | - | 0.5 | 0.094 |
| **Recipient strain** | |  |  |  |  |
| A | regular | 6 | 15 | 0.125 | 0.125 |
| A plasmid 1 | regular | 6 | 60 | 0.19 | 0.19 |
| A plasmid 1 | BZK suppl. | 6 | - | 1.0 | 0.094 |
| B | regular | 6 | 15 | 0.19 | 0.19 |
| B plasmid 2 | regular | 6 | 60 | 0.38 | 0.19 |
| B plasmid 2 | BZK suppl. | 6 | - | 1.0 | 0.094 |
| C | regular | 29 | 15 | 0.094 | 0.064 |
| C plasmid 1 | regular | 29 | 60 | 0.19 | 0.064 |
| C plasmid 1 | BZK suppl. | 29 | - | 1.0 | 0.047 |
|  |  |  |  |  |  |
| *growth medium was supplemented with benzalkonium chloride 30 mg/L | | | |  |  |
| BZK, benzalkonium chloride; mg, milligram; L, litre; MIC, minimum inhibitory concentration; suppl, supplemented. | | | | | |

**Footnote Supplementary Table 4.**

Transformation of naïve strains with plasmid pLMST6 results in phenotypical changes. After transformation, growth was inhibited at benzalkonium chloride concentrations of 60 mg/L, as compared to 15 mg/L before transformation, for all three recipient strains. For gentamicin, minimum inhibitory concentrations increased from 0.125 to 0.19 mg/L, 0.19 to 0.38 mg/L, and 0.094 to 0.19 mg/L on blood agar not supplemented with benzalkonium chloride, and 0.125 to 1.0 mg/L, 0.19 to 1.0 mg/L, and 0.094 to 1.0 mg/L in medium supplemented with benzalkonium chloride. For amoxicillin, minimum inhibitory concentrations before and after transformation were similar on blood agar with or without benzalkonium chloride.
